# Mendelian randomization analysis reveals causal links between specific immune cell phenotypes and Parkinson's disease

**DOI:** 10.1016/j.clinsp.2026.101164

**Published:** 2026-09-22

**Authors:** Yan Su, Sheng Cai, Yang Xu, QiQi Xu, Qi Wang, XianWen Chen

**Affiliations:** Department of Neurology, The First Affiliated Hospital of Anhui Medical University, Hefei City, Anhui Province, China

**Keywords:** Parkinson's disease, Mendelian randomization, Immunophenotyping, Causal inference, Neuroinflammation

## Abstract

**Objective:**

Immunity and neuroinflammation are key factors in Parkinson's Disease (PD); however, detailed studies on how they are causally related to each other are lacking. This study aimed to explore the potential causal relationship between immune cells and PD.

**Methods:**

A comprehensive two-sample Mendelian Randomization (MR) analysis was conducted based on publicly available genetic data to explore the causal relationship between 731 immune cells and PD risk. Inverse Variance Weighted (IVW) served as the main technique to explore the causal relationship, along with methods such as weighted median, MR-Egger regression, simple mode, and weighted mode. Sensitivity analysis was performed to evaluate the reliability, heterogeneity, and horizontal pleiotropy of the study results.

**Results:**

A total of 18 immune cell phenotypes related to PD were identified in MR analysis, with 8 associated with risk, including 3 from the T-cells, B-cells, and Natural Killer cells (TBNK) group, 3 from the regulatory T-cell (Treg) group, and 2 from the myeloid group. There were 10 factors conferring protection against PD, with 3 associated with monocytes, 3 associated with T-cell maturation, 2 associated with Tregs, and 1 associated with myeloid cells. PD altered the phenotypic levels of two immune cell types, one from the TBNK group and one from the B-cell group.

**Conclusions:**

This study suggests a potential causal link between immune cells and PD, pinpointing specific immune cell features related to PD.

## Highlights

• We performed bidirectional two-sample MR of 731 immune cell phenotypes to systematically infer causal associations between immune cells and Parkinson’s disease (PD).
• We identified 18 immune cell phenotypes with causal effects on PD, including 8 risk-related and 10 neuroprotective subtypes.
• Non-classical CD14+CD16+ monocytes were found to reduce the risk of PD, contrasting with findings from previous observational studies and providing new insights into the immune mechanisms underlying PD.

## Background

Parkinson's Disease (PD) is a neurodegenerative disease characterized by the death of dopaminergic neurons in the Substantia Nigra (SN) and the accumulation of α-synuclein (α-syn) protein in Lewy bodies.^1^ Although many pathological processes, such as protein homeostasis imbalance, calcium imbalance, oxidative stress, and chronic inflammation, have been shown to be related to PD,^2^ the pathogenesis of PD remains unclear. Microglia and astrocytes, immune cells that reside in the central nervous system, engage with peripheral immune cells such as macrophages and lymphocytes, triggering a neuroinflammatory response that contributes to PD.^3^

The inflammatory response of immune cells and its importance in PD have received much attention in recent years. A growing body of evidence supports the close relationship between the inflammatory response and immune cells and the onset and progression of brain diseases, including PD.^4^^,^^5^ One study revealed significant features of microglial proliferation and inflammatory response postmortem in the brains of patients with PD.^6^^,^^7^ In addition to involving resident glial cells (microglia and astrocytes) in the central nervous system, this immune response also involves peripheral innate and adaptive immune cells, initiating a systemic immune response.^3^ Infiltrating monocytes, Toll-Like Receptor 4 (TLR4) on monocytes, T cells, and Natural Killer (NK) cells are involved in the midbrain inflammation process and the degeneration of dopamine neurons in PD animal models.^8^^,^^9^ Patients with newly diagnosed PD with a proinflammatory blood environment experience more significant motor impairments and faster cognitive decline.^10^ In PD, the degeneration of the Suprachiasmatic Nucleus (SCN) and the activation of NF-κB and the NLRP3 inflammasome can cause irregular MTN secretion, which normally provides neuroprotection by suppressing inflammation, thereby exacerbating the neuroinflammatory response.^11^^,^^12^ In animal models of PD, targeting modified monocytes has neuroprotective effects.^13^ Moreover, lowering T-cell levels or increasing Treg function provides neuroprotection by mitigating brain inflammation in PD animal models.^14^ Early small-scale safe clinical trials have validated that Sargamostim can transform pro-inflammatory T-cells (Teffs) into a Treg immune response.^15^

Previous observational studies have shown a connection between immune cells and PD, but these studies have several limitations. It is not entirely clear which immune cells contribute to PD pathogenesis and which are considered risk or protective factors. Mendelian Randomization (MR) is a technique based on the independent distribution patterns of Mendelian genetics that uses genetic factors as instrumental variables to greatly eliminate confounding factors and reverse causal bias in observational studies. The natural random classification of genetic variants is the foundation of MR analysis. In line with hereditary principles, parents randomly pass one allele of each gene to their offspring, a process not influenced by confounding factors. Therefore, MR analysis provides a similar approach for randomized controlled trials. For genetic variables to be valid in MR analysis, they must fulfill these three hypotheses: 1) Genetic variation is associated with exposure; 2) Genetic variation is not associated with confounding factors between exposure and outcome; and 3) Genetic variation affects outcome only through exposure.^16^ The final hypothesis is referred to as the no-pleiotropy hypothesis or the exclusion-restriction principle, indicating that genetic variation cannot affect the outcome via alternative causal pathways that do not involve exposure. In two-sample MR analysis, a pair of exposures and outcomes from different or nonoverlapping groups are analyzed, with two-way MR analysis aiming to explore opposite causality.

In this investigation, a two-sample MR analysis was performed on 731 immune cell types and PD to determine the specific causal relationship between immune cells and PD.

## Materials and methods

### Learning planning

This study aims to explore the potential causal relationship between 731 types of immune cells and PD through two-sample MR analysis. MR analysis is guided by three core assumptions: correlation, independence, and exclusion limitations (Fig. 1). For Single-Nucleotide Polymorphisms (SNPs) to be effective as Instrumental Variables (IVs), certain conditions need to be satisfied. The correlation hypothesis proposes that IVs have a strong connection with exposure, such as immune cells, whereas the independence hypothesis posits that IVs are not linked to any confounding factors in the relationship between exposure and outcome. The exclusion limit presumes that the designated IVs influence the outcome (PD) solely through exposure.^17^

**Fig. 1:**
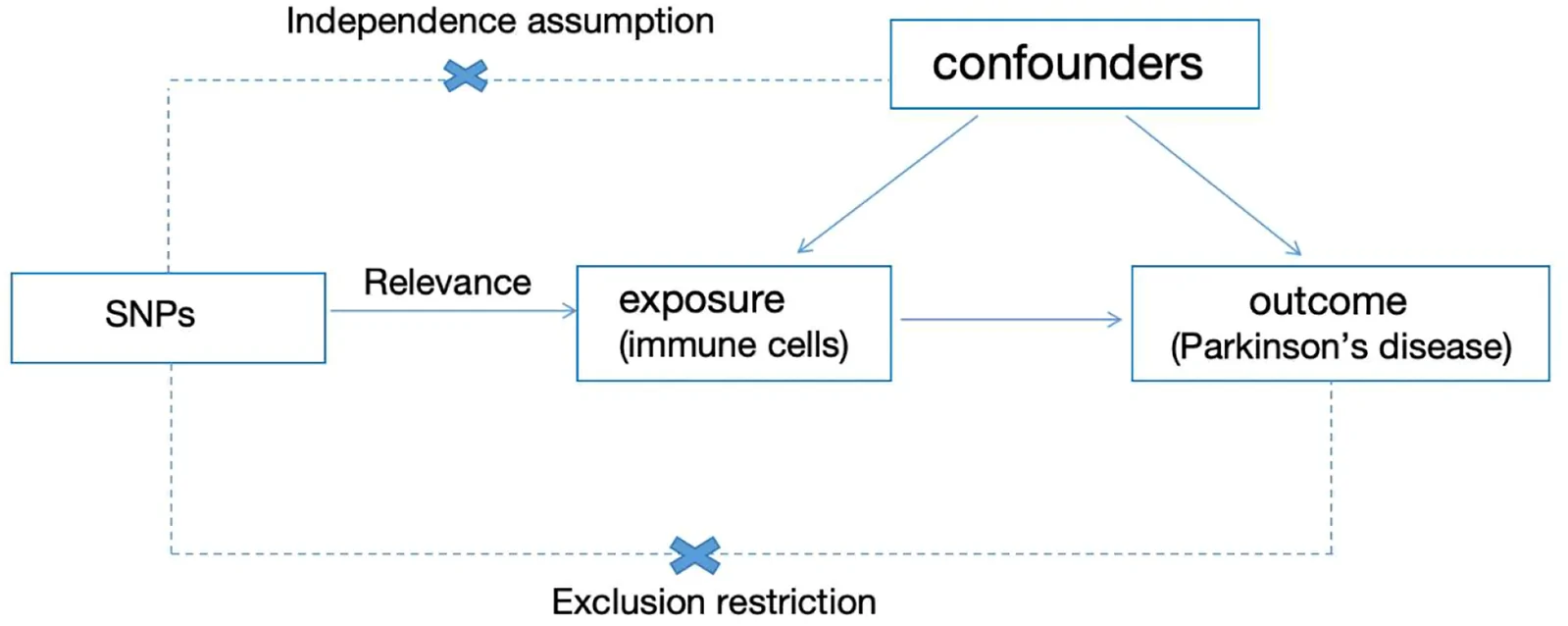
Study design schematic: Mendelian randomization analysis of immune cell phenotypes in patients with PD.

### Data source

The GWAS datasets were used based on public genetic data. This study followed the PRISMA guidelines. The exposure factors included 731 immune cell characteristics (login numbers ranging from GCST0001391 to GCST0002121),^18^ including Median Fluorescence Intensity (MFI), which is indicative of surface antigen levels (n = 389), Relative Cell (RC) count (n = 192), Absolute Cell (AC) count (n = 118), and Morphological Parameters (MP) (n = 32). The features of MFI, AC, and RC include monocytes, B-cells, mature T-cells, TBNK, CDC, bone marrow cells, and Tregs, whereas the features of MP include CDC and TBNK.^17^ The PD dataset was obtained from the GWAS dataset as ebi-a-GCST90018894 (n = 480,018). This study involved 2638 patients of European ancestry with PD and 477,380 controls, and all the studies obtained ethical approval and informed consent.

### Selection of genetic IVs

For the MR hypothesis to hold, there must be a significant correlation between the SNPs chosen as IVs and the exposure. A genome-wide significance threshold of p < 5 × 10^–8^ was used to identify SNPs strongly associated with PD and immune cells. To eliminate bias due to Linkage Disequilibrium (LD), a clustering procedure was used to prune these SNPs (*kb* = 10,000, *r*^2^ = 0.001).^19^^,^^20^ In addition, the F-statistic was applied for strength analysis.^21^ SNPs with an F statistic greater than 10 were deemed sufficiently strong to validate their use as IVs. To further mitigate the concern of weak instrument bias, we calculated the F statistic for each SNP-exposure association, defined as F=(R²/(1−R²))*((N−1−K)/K), where R² is the proportion of variance explained by the SNP, N is the sample size, and *K* is the number of SNPs. The mean *F* statistic across all IVs was [46.3825], with a range from [24.8407] to [137.9650], all of which substantially exceeded the empirical threshold of 10, indicating a low risk of weak instrument bias.

### Statistical analysis

IVW was selected as the main analytical approach. This technique demonstrated peak statistical strength and accuracy in the absence of pleiotropy in IVs.^22^ Furthermore, four additional MR techniques, MR-Egger, Weighted Median (WM), simple mode, and weighted mode, were employed to evaluate the causal link between immune cells and PD. All analyses were conducted using R software (V.4.2.3) for statistical calculations, and the two-sample MR (v.0.5.8) and Mendelian randomization (v.0.9.0) R software packages were used for MR analysis. A forest plot was drawn using the Forestplot package (version 1.1.1). Moreover, False Discovery Rate (FDR) correction was applied for multiple testing, with P_FDR_ < 0.05 considered to indicate statistical significance. However, given the substantial number of traits tested (n = 731), we acknowledge that the FDR procedure, although robust, may not entirely eliminate the risk of false positives. Therefore, we complemented the FDR criterion by emphasizing the consistency of effect estimates across different MR methods and the biological plausibility of the findings when interpreting the results.

### Sensitivity analysis

Sensitivity analysis was performed to guarantee the robustness of the findings. We conducted multiple analyses to rigorously evaluate potential violations of the MR assumptions, particularly horizontal pleiotropy. Initially, the MR-Egger intercept test was used to assess pleiotropy among genetic diversity and other possible confounding elements, and the variance between the regression intercept and zero was analyzed to identify horizontal pleiotropy. A nonsignificant intercept (p > 0.05) suggests that directional pleiotropy does not bias the MR results.^23^ The heterogeneity of SNPs within the IVW and MR-Egger techniques was evaluated through Cochran's *Q*-test, where a p-value exceeding 0.05 signifies a lack of heterogeneity.^24^ Although heterogeneity does not necessarily imply pleiotropy, its presence warrants the use of robust MR methods. The MR-PRESSO method was employed to detect any anomalies^25^ (Supplementary Table 1). This method identifies and corrects for outliers driven by horizontal pleiotropy, providing a pleiotropy-corrected causal estimate. Following this, the leave-one-out technique was employed to evaluate how individual SNPs influence causal effect calculations, aiming to identify possible biases or confounding elements^26^ (Supplementary Figs. 1–3). Both scatter plots and funnel plots were utilized as well. Outliers did not affect the results shown in the scatter plots. The funnel graph illustrates the strong correlation and the lack of heterogeneity. The overall consistency of the results across multiple MR methods and the absence of significant pleiotropy in these sensitivity analyses strengthen the validity of our causal inferences.

## Results

### Causal effect of immune cells on PD

#### Risk factor

The impact of immune cell phenotypes on PD was explored using IVW, weighted median, MR-Egger regression, simple mode, and weighted mode (Fig. 2). By employing IVW as the primary analysis technique and applying multiple tests with FDR adjustments, immune characteristics were pinpointed at a 0.05 significance level, revealing 8 immunophenotypes associated with an increased risk of PD (Fig. 3). Among them, 3 were in the TBNK group (HLA DR++monocyte% monocytes, CD45 on CD14+ monocytes, and CD45 on HLA DR+ T-cells), 3 were in the Treg group (CD28+DN (CD4^‒^CD8^‒^)% T-cells, activated Treg% CD4 Tregs, and CD39 on monocytes), and 2 were in the myeloid group (CD33dim HLA DR+CD11b+AC and CD33br HLA DR+CD14-AC). IVW analysis revealed a significant positive correlation (OR = 1.11; 95% CI 1.02–1.20, p = 0.0139) between elevated levels of monocytic human leukocyte antigen HLA DR++ monocyte% monocytes (TBNK group) and an increased risk of PD. Moreover, increased CD45 expression on CD14+ monocytes (TBNK group) (OR = 1.11, 95% CI 1.02–1.21, p = 0.0208), elevated CD45 on HLA DR+ T-cells (TBNK group) (OR = 1.06, 95% CI 1.00–1.13, p = 0.0476), elevated CD28+DN (CD4^-^CD8^‒^)% T-cells (Treg group) (OR = 1.10, 95% CI 1.02–1.18, p = 0.0193), elevated activated Treg% CD4 Tregs (Treg group) (OR = 1.07, 95% CI 1.01–1.13, p = 0.0203), elevated CD39 on monocytes (Treg group) (OR = 1.03, 95% CI 1.00–1.06, p = 0.0454), elevated CD33dim HLA DR+CD11b+AC (myeloid cell group) (OR = 1.08, 95% CI 1.02–1.14, p = 0.0076), and elevated CD33br HLA DR^+^CD14^-^ AC (myeloid cell group) (OR = 1.03, 95% CI 1.00–1.05, p = 0.0482) showed positive correlations with increased risk of PD. No heterogeneity was detected by Cochran's *Q*-test, and the MR-Egger intercept test did not reveal any pleiotropy (Table 1, Supplementary Tables 2–4). Scatter and funnel plots also confirmed the stability of the results (Supplementary Figs. 4–5).

**Fig. 2:**
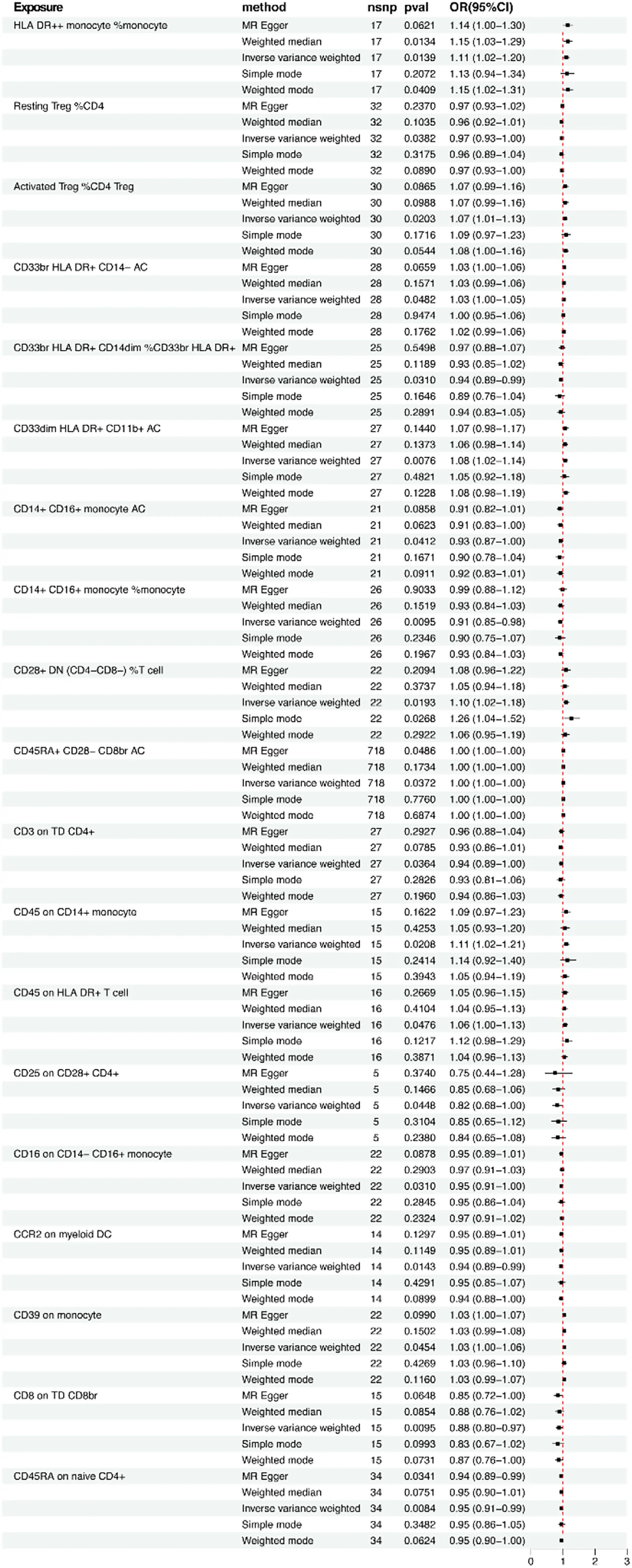
Forest plot of the MR analysis for the causal effect of immunophenotype on PD using multiple MR methods. MR, Mendelian Randomization; PD, Parkinson's Disease; OR, Odds Ratio; 95% CI, 95% Confidence Interval.

**Fig. 3:**
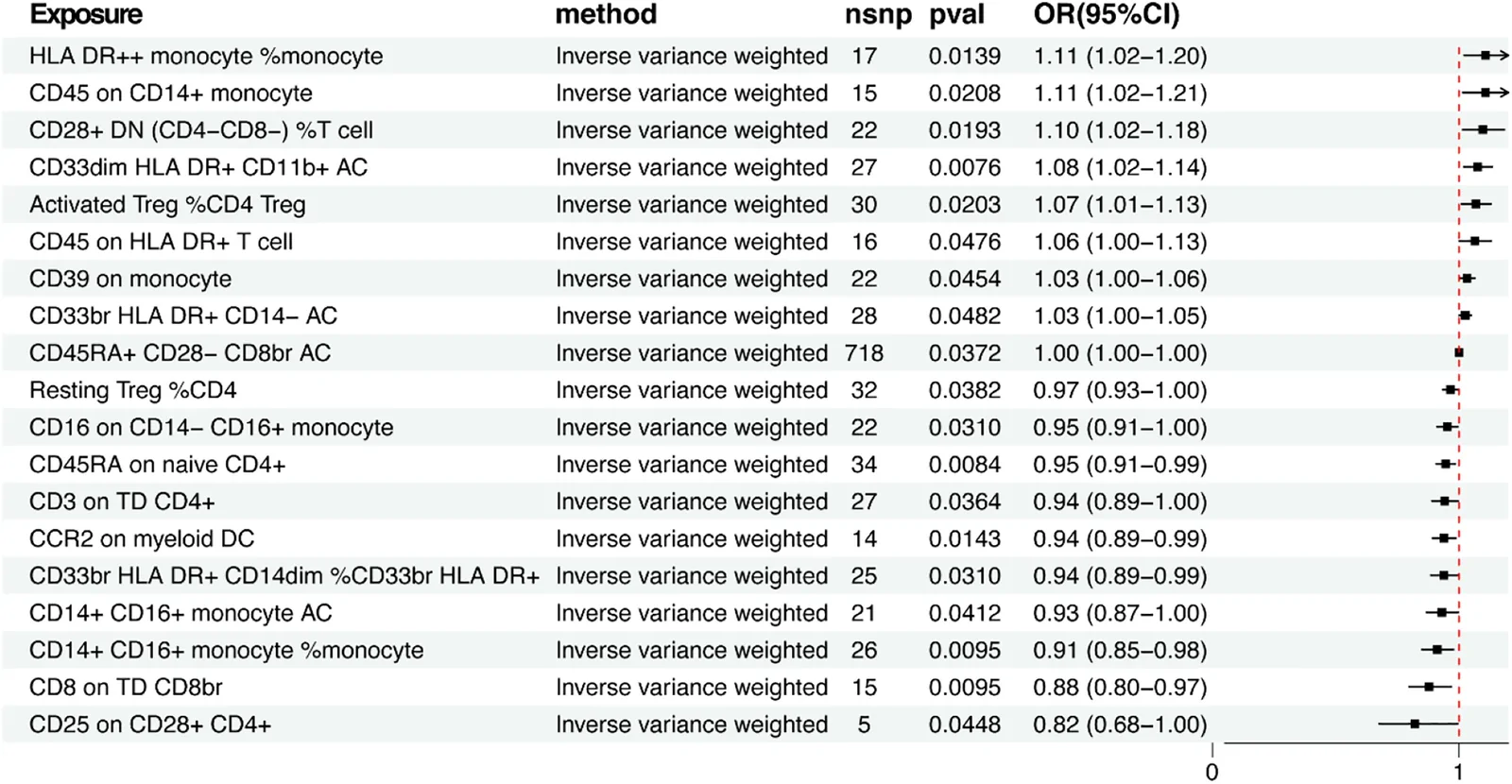
Forest plot of the MR analysis for the causal effect of immunophenotype on PD using the IVW method. MR, Mendelian Randomization; PD, Parkinson's Disease; IVW, Inverse Variance-Weighted; OR, Odds Ratio; 95% CI, 95% Confidence Interval.

**Table 1:** Heterogeneity and pleiotropy test (risk factor).

Exposure	Outcome	IVW OR (95% CI)	IVW p-value	Heterogeneity (Q_pval)	Pleiotropy (Egger pval)
HLA DR++ monocyte %monocyte	PD	1.11 (1.02‒1.20)	0.0139	0.278	0.550
CD45 on CD14+ monocyte	PD	1.11 (1.02‒1.21)	0.0208	0.553	0.769
CD45 on HLA DR+ T-cell	PD	1.06 (1.00‒1.13)	0.0476	0.580	0.804
CD28+ DN (CD4−CD8−) %T-cell	PD	1.10 (1.02‒1.18)	0.0193	0.395	0.802
Activated Treg %CD4 Treg	PD	1.07 (1.01‒1.13)	0.0203	0.866	0.851
CD39 on monocyte	PD	1.03 (1.00‒1.06)	0.0454	0.879	0.947
CD33dim HLA DR+ CD11b+ AC	PD	1.08 (1.02‒1.14)	0.0076	0.365	0.935
CD33br HLA DR+ CD14− AC	PD	1.03 (1.00‒1.05)	0.0482	0.643	0.653

#### Protective factor

A total of 10 immunophenotypes showing protective effects on PD (Fig. 3) were identified, including 3 in the monocyte group (CD14+CD16+monocyte AC, CD16 on CD14^−^CD16+ monocytes, and CD14+CD16+monocyte% monocytes), 3 in the T-cell maturation stage group (CD8 on TD CD8br, CD45RA on naive CD4+, and CD3 on TD CD4+), 2 in the Treg group (resting Treg% CD4 and CD25 on CD28+CD4+), 1 in the CDC group (CCR2 on myeloid DCs), and 1 in the myeloid cell group (CD33br HLA DR+CD14dim% CD33br HLA DR+). IVW results revealed CD16 on CD14^−^CD16+ monocytes (monocyte group) (OR = 0.95, 95% CI 0.91–1.00, p = 0.0310), CD14+CD16+monocyte AC (monocyte group) (OR = 0.93, 95% CI 0.87–1.00, p = 0.0412), CD14+CD16+monocyte% monocytes (monocyte group) (OR = 0.91, 95% CI 0.85–0.98, p = 0.0095), CD45RA on naive CD4+ cells (T-cell maturation stages) (OR = 0.95, 95% CI 0.91–0.99, p = 0.0084), CD8 on TD CD8br (T-cell maturation stages) (OR = 0.88, 95% CI 0.80–0.97, p = 0.0095), CD3 on TD CD4+ cells (T-cell maturation stages) (OR = 0.94, 95% CI 0.89–1.00, p = 0.0364), resting Treg% CD4 (Treg group) (OR = 0.97, 95% CI 0.93–1.00, p = 0.0382), CD25 on CD28+CD4+ (Treg group) (OR = 0.82, 95% CI 0.68–1.00, p = 0.0448), CCR2 on myoid DCs (CDC group) (OR = 0.94, 95% CI 0.89–0.99, p = 0.0143), and CD33br HLA DR+CD14dim% CD33br HLA DR+ (myeloid cell group) (OR = 0.94, 95% CI 0.89–0.99, p = 0.0310) suggested an association with a reduced risk of PD. Cochran’s *Q*-test revealed no heterogeneity, and the MR-Egger intercept test failed to reveal horizontal pleiotropy (Supplementary Tables 5–7). Both the scatter and funnel plots corroborated the stability of the findings (Supplementary Figs. 6–7).

### The impact of PD on immune cell characteristics

Reverse MR analysis was conducted (Fig. 4), with the IVW method as the main method (Fig. 5). Two immune cell phenotypes were discovered (p < 0.05). The occurrence of PD was negatively correlated with HLA-DR+ T-cell AC (TBNK group) (OR = 0.91, 95% CI 0.83–0.99, p = 0.0325) and IgD-CD38% B-cells (B-cell group) (OR = 0.88, 95% CI 0.80–0.98, p = 0.0175). According to the results of the Cochran’s *Q*-test, there was no heterogeneity, and the MR-Egger intercept test did not reveal horizontal pleiotropy (Supplementary Table 8). The stability of the results was also confirmed by the scatter and funnel plots (Fig. 6, Fig. 7).

**Fig. 4:**
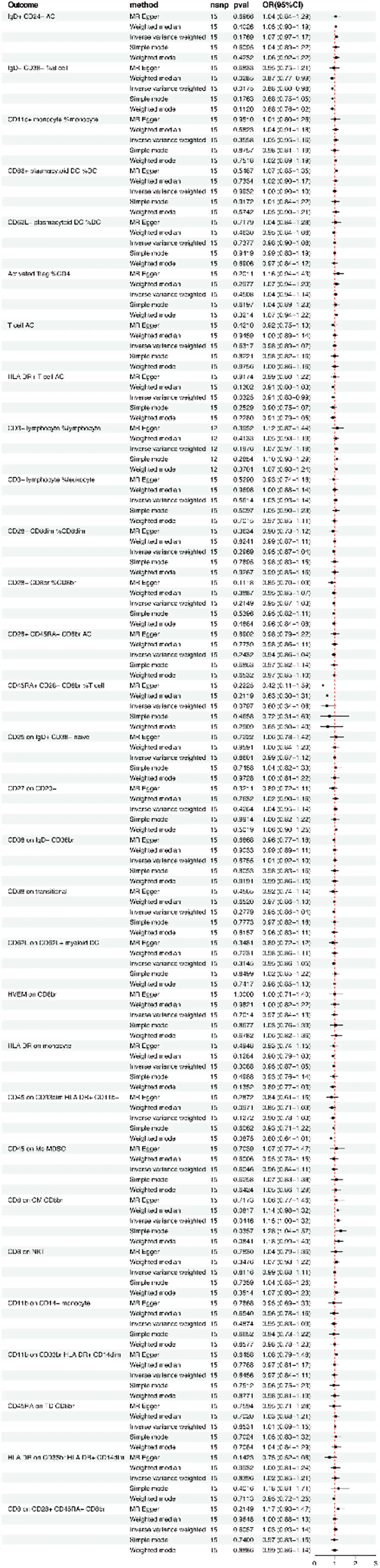
Forest plot of the reverse MR analysis for the causal effect of PD on immunophenotypes using multiple MR methods. MR, Mendelian Randomization; PD, Parkinson's Disease; OR, Odds Ratio; 95% CI, 95% Confidence Interval.

**Fig. 5:**
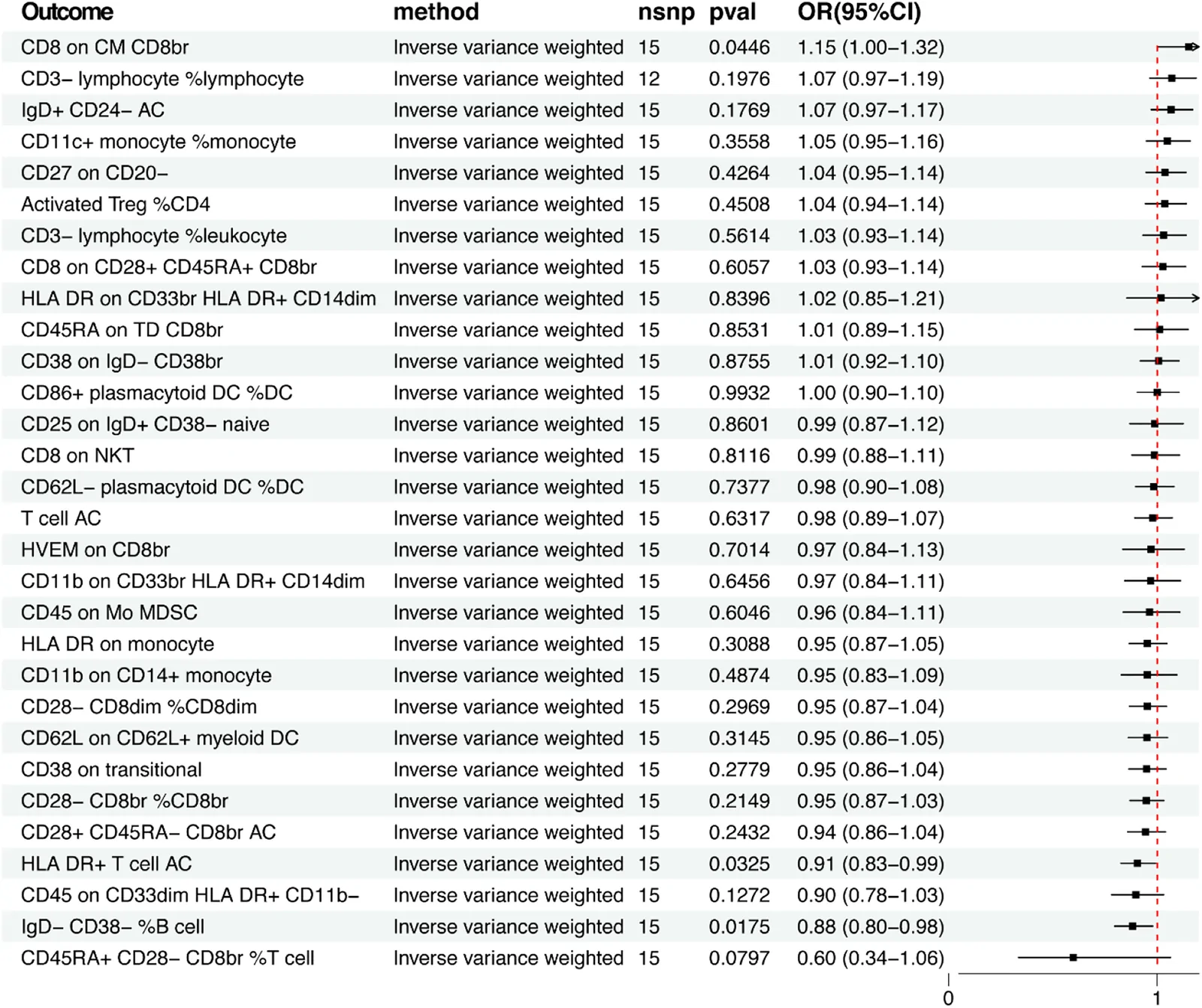
Forest plot of the reverse MR analysis for the causal effect of PD on immunophenotypes using the IVW method. MR, Mendelian Randomization; PD, Parkinson's Disease; IVW, Inverse Variance-Weighted; OR, Odds Ratio; 95% CI, 95% Confidence Interval.

**Fig. 6:**
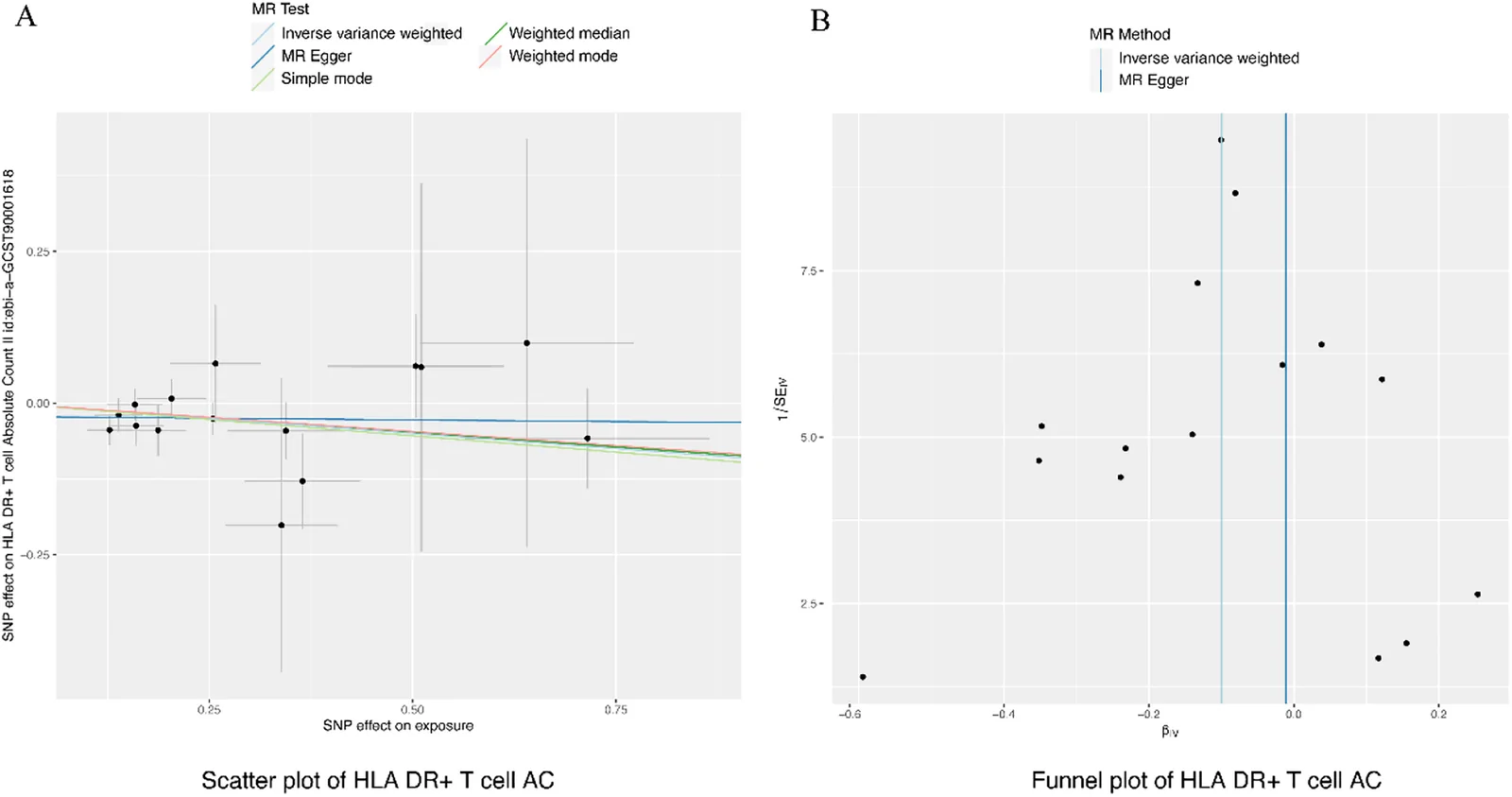
Scatter and funnel plots of HLA-DR+ T-cell AC. (A) Scatter plot of HLA-DR+T-cell AC; (B) funnel plot of HLA-DR+T-cell AC.

**Fig. 7:**
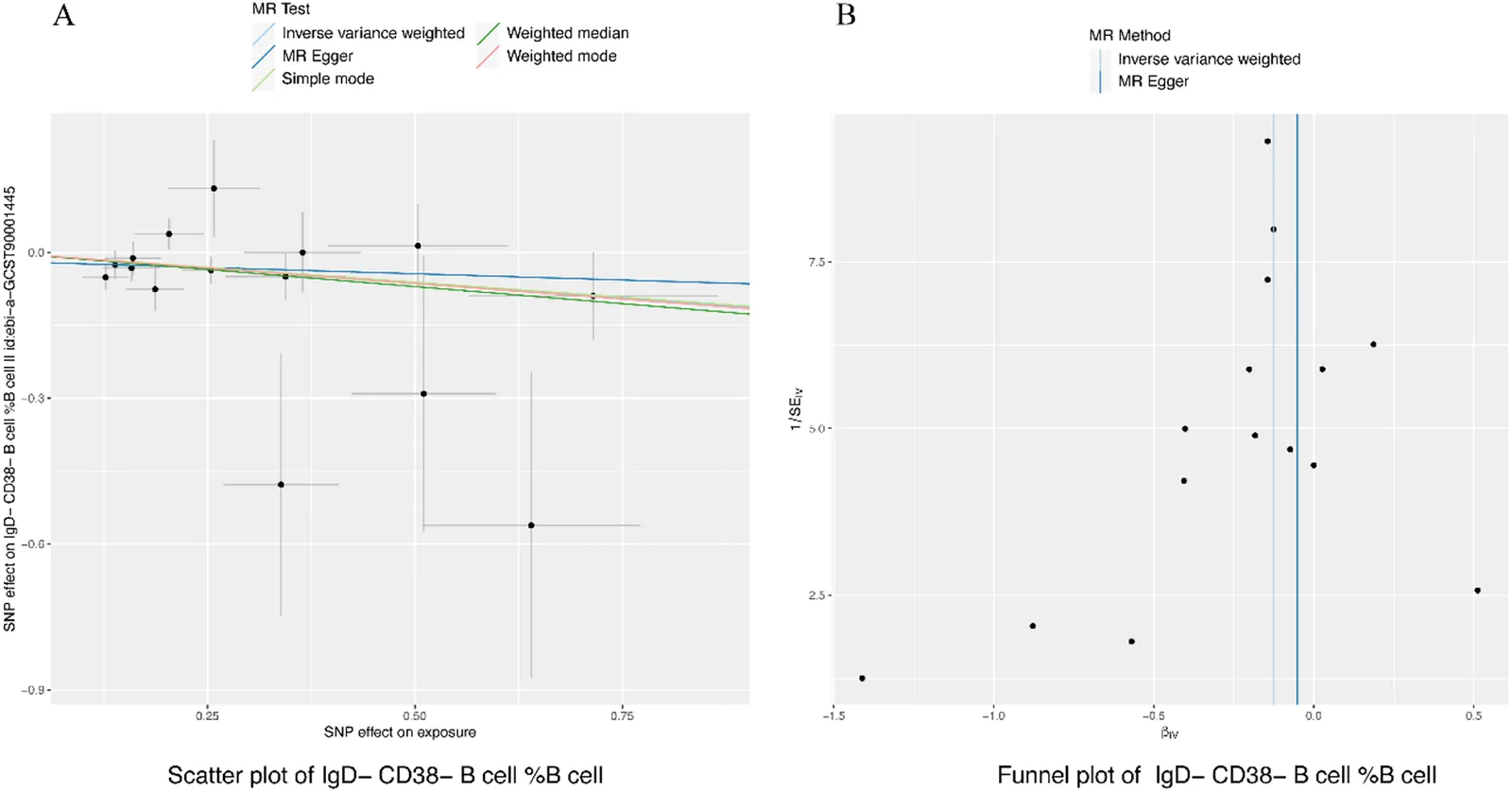
Scatter and funnel plots of IgD-CD38+ B cells. (A) Scatter plot of IgD-CD38+ B cells; (B) funnel plot of IgD-CD38+ B cells.

In the reverse MR analysis, which assessed the potential effect of PD on immune phenotypes, only two significant associations were identified. This limited number of findings may reflect a true absence of strong causal effects from PD on the immune system, but it could also be attributable to a lack of statistical power for such bidirectional inference, particularly given the smaller sample sizes of the immune trait GWAS than those of the PD GWAS.

## Discussion

Previous observational studies have demonstrated a correlation between immune cells and PD.^27^ Nonetheless, the limitations of these studies mean that the relationship between immune cells and PD is still not well understood. This research employed two-sample MR analysis to investigate the causal link between 731 immune cell types and PD. PD was influenced by 18 different immune cell types, including 8-risk elements and 10 protective elements associated with monocytes, TBNK, Tregs, myeloid cells, T-cell maturation stages, and CDC. In the reverse analysis, PD was negatively correlated with HLA DR+T-cell AC (TBNK group) and IgD-CD38^−^% B-cells (B-cell group).

This study revealed that elevated CD14+CD16+monocyte% monocytes, CD14+CD16+monocyte AC, and CD16 on CD14^−^CD16+ monocytes were associated with a reduced risk of PD. A whole-epigenome association study of peripheral immune cell populations in PD revealed six differentially methylated cytosine phosphate guanines in CD14+ monocytes, which are closely related to the onset of PD.^28^ In PD queue research, there is a marked increase in monocytes in the peripheral blood and cerebrospinal fluid among patients with PD.^29^ The increase in monocytes in blood samples from patients with PD is positively correlated with the severity of PD.^30^ Changes in monocytes in the blood are more significant in patients with PD with cognitive impairment.^29^ Most previous observational studies have reported a positive correlation between increased monocyte levels and a higher risk of PD. In contrast, our MR analysis revealed that CD16 expression on CD14^−^CD16+ monocytes, CD14+CD16+ monocyte AC, and CD14+CD16+monocyte% as protective factors against PD. This apparent discrepancy may stem from key methodological differences: whereas observational studies can be influenced by confounding or disease-induced changes, MR captures lifelong genetic effects that are immune to reverse causation. Biologically, the protective role suggested by our findings might be attributed to the specific functions of certain monocyte subsets ‒ such as their ability to patrol the vascular endothelium and contribute to vascular and tissue protection and repair.^31^ Moreover, receptors such as CD14 increase the capacity of monocytes to recognize and clear pathological proteins (e.g., aggregated α-synuclein) and respond to invasive pathogens,^32^ potentially indicating a protective clearance mechanism in PD. Nevertheless, the complex and dual roles of innate immunity in neurodegeneration warrant further investigation to fully elucidate the specific functions of these monocyte subsets in PD pathogenesis.

As a category of white blood cells, lymphocytes have specialized immune functions, mainly comprising T-lymphocytes for cellular immunity, B-lymphocytes for humoral immunity, and NK cells capable of directly killing target cells. Maintaining normal immune function requires an appropriate balance of TBNK in the body.^33^ MR analysis revealed that increased HLA DR++monocyte% monocytes, CD45 on CD14+ monocytes, and CD45 on HLA DR+T-cells of the TBNK group were closely related to the risk of PD. In addition, the occurrence of PD caused decreases in HLA-DR+ T-cell AC (TBNK group) and IgD-CD38^−^ B-cell levels (B-cell group). B-lymphocytes in the peripheral blood of patients with PD were significantly reduced, especially in patients with PD and dementia.^34^ In an animal model of PD, a decrease in B-cells was related to poor pathology and behavior.^34^ In patients with PD, B cells have an improved ability to present antigens and become activated, contributing to neuroprotection.^35^ To advance clinical trials, the next step is to perform animal experiments to determine whether changing these B-cells can affect disease outcomes.^36^ A significant number of drugs are available that target B-lymphocytes, allowing for their inclusion in trials once the correct targets are clearly identified.

Research has shown that mature T-cells and Tregs are closely related to PD. T-cells are involved in the inflammatory response and in the death of dopaminergic neurons in the PD brain. Tregs, which are essential for immune regulation, help control inflammation. In PD animal models, either reducing T-cells or increasing Treg capabilities is neuroprotective.^14^ Cell therapy with Tregs has therapeutic potential in PD mouse models, as α-Syn inhibits the inflammatory response in PD by interacting with Tregs and has a protective effect.^37^ In addition, T-cell-driven inflammation is a gut-brain axis mediator involved in PD. During gut microbiota dysbiosis, T-cells mediate pathological changes in the gut microbiota, resulting in gut barrier disruption and changes in blood-brain barrier permeability, which in turn lead to neuroinflammatory responses in the brain.^38^ The two-sample MR study revealed that CD45RA expression on naive CD4+ cells, CD8 expression on TD CD8br cells, and CD3 expression on TD CD4+ cells during the T-cell maturation stages and Resting Treg% CD4, CD25 on CD28+CD4+ cells in the Treg group correlated with a lower risk of PD and served as defensive elements against PD. Elevated levels of CD28+DN (CD4^−^CD8^−^) T-cells, Treg% CD4 Tregs, and CD39 on monocytes were associated with an increased risk of PD. These T-cells may be closely related to the pathogenesis of PD because they participate in the inflammatory response of the gut-brain axis.

Previous research has revealed a significant connection between myeloid cells and neuroinflammation in PD, making it a focus for treatment.^39^ This study revealed a link between CD33dim HLA DR+CD11b+ AC and CD33br HLA DR+CD14^−^ AC in myeloid cells and a heightened risk of PD, whereas CCR2 expression in myoid DCs within CDC cells correlated with decreased PD risk. CD33br HLA DR+CD14dim% and CD33br HLA DR+ were protective factors against PD.

Our MR analysis revealed 18 immune cell traits that were significantly associated with PD. It is important to note, however, that the Odds Ratios (ORs) for some of these significant associations were close to 1. Although these estimates are statistically robust, they indicate that per standard deviation change in immune cell traits, the effect on the log odds of PD risk or protection is very small. Such minimal effects are consistent with the polygenic and multifactorial nature of PD, where individual biological factors typically contribute only minimal incremental risk or protective effects. The clinical translation of individual effects of this magnitude is likely limited; however, they remain valuable for understanding the aggregated biological pathways and etiological architecture of the disease. Furthermore, several associations with borderline p values in our analysis should be considered preliminary and hypothesis-generating. These findings warrant further investigation in larger, independent cohorts to confirm their validity.

Reverse MR analysis revealed only two significant associations, suggesting that PD genetic liability may have a limited direct causal effect on most of the immune phenotypes investigated here. However, the reverse MR analysis had lower statistical power, partly due to the disparity in sample sizes between the typically smaller immune GWAS and the larger PD consortium. Therefore, we cannot rule out the possibility that PD has more subtle, widespread effects on the immune system that larger future studies may uncover. These results primarily highlight the dominance of immune-to-PD causal pathways in our current data; however, the bidirectional relationship warrants further investigation.

In summary, our MR analysis revealed peripheral immune signatures derived from blood-based immunophenotypes as potential causal factors influencing PD risk. These findings contribute to clinical practice by informing treatment selection and prognosis evaluation for patients with PD while also offering new directions for drug development. Importantly, whether these systemic immune effects involve Central Nervous System (CNS) mechanisms, such as microglial activation or neuroinflammation, remains unclear and warrants further investigation. This distinction is critical for clinical interpretation, particularly when considering therapies that target peripheral versus central immune pathways. Furthermore, the generalizability of these causal inferences to non-European populations (e.g., African, East Asian, or Hispanic ancestries) cannot be assumed due to potential differences in genetic architecture, linkage disequilibrium patterns, and environmental exposures. Notably, the statistical power of our study was primarily sufficient to detect associations with moderate to large effect sizes. The relatively limited sample size for patients with PD may have constrained our ability to identify genuine but weaker genetic associations. Additionally, the diverse clinical characteristics of different immune cells implicated in PD were clearly observable. Consequently, the anticipated outcomes might not consistently emerge from just one therapy. Additional studies are needed to investigate the dynamics between patients with PD and immune cells.

### Strengths

This study has several advantages. First, this study investigated the association between immune cell phenotypic characteristics and PD risk, which could help researchers identify potential drug targets for PD. This method addresses the drawbacks of observational studies and sidesteps the time and resource demands of randomized controlled trials. Second, this study could aid in the development of personalized prevention and treatment strategies for PD, and intervention in the early stages of the disease could improve the quality of life for people with PD. Third, unlike previous studies of single cell types, different immune cell types were covered in this study. Fourth, different sensitivity analyses were conducted to ensure reliability and consistency.

### Limitation

This study has several limitations. First, despite FDR correction, the analysis of 731 immune traits revealed a risk of false positives, and results with borderline significance should be interpreted cautiously. Second, many associations had odds ratios close to 1, indicating statistically robust but biologically modest effects. Although consistent with the polygenic nature of PD, these findings emphasize etiological insights over direct clinical translatability. Third, although sensitivity analyses (MR-Egger, MR-PRESSO) revealed no significant pleiotropy, residual bias due to horizontal pleiotropy cannot be fully excluded. Fourth, the restriction to populations of European ancestry limits generalizability, highlighting the need for future studies in diverse cohorts. Fifth, although MR provides evidence for causality, it does not replace the need for functional validation or clinical trials. Further mechanistic and translational studies are essential to clarify the precise role of immune cells in PD pathogenesis. Finally, when these results are interpreted, the clinical heterogeneity of PD must be considered. Our MR analysis uses genetic instruments derived from broad PD GWASs, which do not capture disease subtypes or stages. Therefore, our findings reflect average effects on overall PD risk, and their relevance to specific clinical presentations requires future investigations using subtype-stratified data.

## Conclusion

In summary, this Mendelian randomization study identified several potential causal associations between immune cell phenotypes and PD, thereby providing genetic evidence that contributes to our understanding of the neuroimmune axis in PD. As these findings are exploratory and hypothesis-generating, they highlight the need for future research to first independently replicate these associations and then elucidate their underlying biological mechanisms, which may ultimately contribute to the long-term goal of identifying novel therapeutic targets.

## Ethics statement

Our study is a secondary analysis of publicly available GWAS summary data and does not involve individual-level human data; therefore, no separate ethics approval or ethics ID applies to this study. All original GWAS studies obtained ethical approval and informed consent from participants, as clearly stated in our manuscript.

## Ethics approval and consent to participate

This analysis is based on publicly sourced GWAS summary statistics. Each contributing study secured ethical approval and participant consent during its original conduct.

## Funding

The present study was supported by the 10.13039/501100001809National Natural Science Foundation of China (Grant number: 81971072).

## Data availability statement

The data that support the findings of this study are available from the corresponding author upon reasonable request. Authors confirm that they have the right to share the summarized data used in the MR analysis.

## Conflicts of interest

The authors declare no conflicts of interest.

## References

[1] Bloem B.R., Okun M.S., Klein C. (2021). Parkinson's disease. Lancet.

[2] Morris H.R., Spillantini M.G., Sue C.M. (2024). Williams-gray CH. The pathogenesis of Parkinson's disease. Lancet.

[3] Tansey M.G., Wallings R.L., Houser M.C., Herrick M.K., Keating C.E. (2022). Joers V. Inflammation and immune dysfunction in Parkinson disease. Nat Rev Immunol.

[4] López-Cerdán A., Andreu Z., Hidalgo M.R., Grillo-Risco R., Català-Senent J.F., Soler-Sáez I. (2022). Unveiling sex-based differences in Parkinson's disease: a comprehensive meta-analysis of transcriptomic studies. Biol Sex Differ.

[5] Carlisle S.M., Qin H., Hendrickson R.C., Muwanguzi J.E., Lefkowitz E.J., Kennedy R.E. (2021). Sex-based differences in the activation of peripheral blood monocytes in early Parkinson disease. NPJ Parkinsons Dis.

[6] Singh D. (2022). Astrocytic and microglial cells as the modulators of neuroinflammation in Alzheimer's disease. J Neuroinflamm.

[7] Croisier E., Moran L.B., Dexter D.T., Pearce R.K., Graeber M.B. (2005). Microglial inflammation in the parkinsonian substantia nigra: relationship to alpha-synuclein deposition. J Neuroinflamm.

[8] Karikari A.A., McFleder R.L., Ribechini E., Blum R., Bruttel V., Knorr S. (2022). Neurodegeneration by α-synuclein-specific T cells in AAV-A53T-α-synuclein Parkinson's disease mice. Brain Behav Immun.

[9] Farmen K., Nissen S.K., Stokholm M.G., Iranzo A., Østergaard K., Serradell M. (2021). Monocyte markers correlate with immune and neuronal brain changes in REM sleep behavior disorder. Proc Natl Acad Sci U S A.

[10] CH Williams-Gray, Wijeyekoon R., Yarnall A.J., Lawson R.A., Breen D.P., Evans J.R. (2016). Serum immune markers and disease progression in an incident Parkinson's disease cohort (ICICLE-PD). Mov Disord.

[11] de Lima E., Soares J.M., del Carmen Sanabria Garrido Y., Gomes Valente S., Priel M.R., Chada Baracat E. (2005). Effects of pinealectomy and the treatment with melatonin on the temporal lobe epilepsy in rats. Brain Res.

[12] Turkistani A., Al-Kuraishy H.M., Al-Gareeb A.I., Negm W.A., Bahaa M.M., Metawee M.E. (2024). Melatonin circadian rhythm in Parkinson's disease: express bewilderment. Neurotox Res.

[13] Ferreira S.A., Li C., Klæstrup I.H., Vitic Z., Rasmussen R.K., Kirkegaard A. (2023). Sex-dimorphic neuroprotective effect of CD163 in an α-synuclein mouse model of Parkinson's disease. NPJ Parkinsons Dis.

[14] Williams G.P., Schonhoff A.M., Jurkuvenaite A., Gallups N.J., Standaert D.G., Harms A.S. (2021). CD4 T cells mediate brain inflammation and neurodegeneration in a mouse model of Parkinson's disease. Brain.

[15] Olson K.E., Abdelmoaty M.M., Namminga K.L., Lu Y., Obaro H., Santamaria P. (2023). An open-label multiyear study of sargramostim-treated Parkinson's disease patients examining drug safety, tolerability, and immune biomarkers from limited case numbers. Transl Neurodegener.

[16] Zhu G.L., Xu C., Yang K.B., Tang S.Q., Tang L.L., Chen L. (2022). Causal relationship between genetically predicted depression and cancer risk: a two-sample bi-directional mendelian randomization. BMC Cancer.

[17] Wang C., Zhu D., Zhang D., Zuo X., Yao L., Liu T. (2023). Causal role of immune cells in schizophrenia: mendelian randomization (MR) study. BMC Psychiatry.

[18] Orrù V., Steri M., Sidore C., Marongiu M., Serra V., Olla S. (2020). Complex genetic signatures in immune cells underlie autoimmunity and inform therapy. Nat Genet.

[19] Hemani G., Zheng J., Elsworth B., Wade K.H., Haberland V., Baird D. (2018). The MR-base platform supports systematic causal inference across the human phenome. Elife.

[20] Auton A., Brooks L.D., Durbin R.M., Garrison E.P., Kang H.M., Korbel J.O. (2015). A global reference for human genetic variation. Nature.

[21] Mensah-Kane J., Schmidt A.F., Hingorani A.D., Finan C., Chen Y., van Duijvenboden S. (2021). No clinically relevant effect of heart rate increase and heart rate recovery during exercise on cardiovascular disease: a mendelian randomization analysis. Front Genet.

[22] Xue H., Shen X., Pan W. (2021). Constrained maximum likelihood-based Mendelian randomization robust to both correlated and uncorrelated pleiotropic effects. Am J Hum Genet.

[23] Bowden J., Davey Smith G., Burgess S. (2015). Mendelian randomization with invalid instruments: effect estimation and bias detection through Egger regression. Int J Epidemiol.

[24] Greco M.F., Minelli C., Sheehan N.A., Thompson J.R. (2015). Detecting pleiotropy in Mendelian randomisation studies with summary data and a continuous outcome. Stat Med.

[25] Verbanck M., Chen C.Y., Neale B., Do R. (2018). Detection of widespread horizontal pleiotropy in causal relationships inferred from Mendelian randomization between complex traits and diseases. Nat Genet.

[26] Burgess S., Davey Smith G., Davies N.M., Dudbridge F., Gill D., Glymour M.M. (2019). Guidelines for performing mendelian randomization investigations: update for summer 2023. Wellcome Open Res.

[27] Lauritsen J., Romero-Ramos M. (2023). The systemic immune response in Parkinson's disease: focus on the peripheral immune component. Trends Neurosci.

[28] Andersen M.S., Leikfoss I.S., Brorson I.S., Cappelletti C., Bettencourt C., Toft M. (2023). Epigenome-wide association study of peripheral immune cell populations in Parkinson's disease. NPJ Parkinsons Dis.

[29] Wijeyekoon R.S., Kronenberg-Versteeg D., Scott K.M., Hayat S., Kuan W.L., Evans J.R. (2020). Peripheral innate immune and bacterial signals relate to clinical heterogeneity in Parkinson's disease. Brain Behav Immun.

[30] Thome A.D., Atassi F., Wang J., Faridar A., Zhao W., Thonhoff J.R. (2021). Ex vivo expansion of dysfunctional regulatory T lymphocytes restores suppressive function in Parkinson's disease. NPJ Parkinsons Dis.

[31] Roodveldt C., Bernardino L., Oztop-Cakmak O., Dragic M., Fladmark K.E., Ertan S. (2024). The immune system in Parkinson's disease: what we know so far. Brain.

[32] Devitt A., Moffatt O.D., Raykundalia C., Capra J.D., Simmons D.L., Gregory C.D. (1998). Human CD14 mediates recognition and phagocytosis of apoptotic cells. Nature.

[33] Hong X., Xiao Z. (2023). Changes in peripheral blood TBNK lymphocyte subsets and their association with acute exacerbation of chronic obstructive pulmonary disease. J Int Med Res.

[34] Scott K.M., Chong Y.T., Park S., Wijeyekoon R.S., Hayat S., Mathews R.J. (2023). B lymphocyte responses in Parkinson's disease and their possible significance in disease progression. Brain Commun.

[35] Scott KM.B (2022). Lymphocytes in Parkinson's disease. J Parkinsons Dis.

[36] Wang P., Luo M., Zhou W., Jin X., Xu Z., Yan S. (2022). Global characterization of peripheral B Cells in Parkinson's disease by single-cell RNA and BCR sequencing. Front Immunol.

[37] Huang P., Zhang Z., Zhang P., Feng J., Xie J., Zheng Y. (2024). TREM2 deficiency aggravates NLRP3 inflammasome activation and Pyroptosis in MPTP-induced Parkinson's disease mice and LPS-induced BV2 cells. Mol Neurobiol.

[38] Campos-Acuña J., Elgueta D., Pacheco R. (2019). T-cell-driven inflammation as a mediator of the gut-brain axis involved in Parkinson's. Disease Front Immunol.

[39] Wang B., Ma Y., Li S., Yao H., Gu M., Liu Y. (2023). GSDMD in peripheral myeloid cells regulates microglial immune training and neuroinflammation in Parkinson's disease. Acta Pharm Sin B.

