# Supplementary material for: Mendelian randomization analysis reveals causal links between specific immune cell phenotypes and Parkinson's disease
Source: Clinics (Sao Paulo). 2026 Sep 22;81:101164. doi: 10.1016/j.clinsp.2026.101164 (PMC13636344; doi:10.1016/j.clinsp.2026.101164)
Supplement: Supplementary file 1 [file mmc1.docx]

**CLINICS-D-24-00564_Material Supplementary**

**Supplementary Figure 1**. Leave-one-out analysis result of immunophenotypes on PD (Risk factor).

(A)HLA DR++ monocyte %monocyte; (B) CD45 on CD14+ monocyte; (C) CD45 on HLA DR+ T cell; (D) CD28+ DN (CD4−CD8−) %T cell; (E) Activated Treg %CD4 Treg; (F) CD39 on monocyte; (G) CD33dim HLA DR+ CD11b+ AC; (H) CD33br HLA DR+ CD14− AC.

**Supplementary Figure 2**. Leave-one-out analysis result of immunophenotypes on PD (Protective factor). (A) CD16 on CD14− CD16+ monocyte; (B) CD14+ CD16+ monocyte AC; (C) CD14+ CD16+ monocyte %monocyte; (D)CD45RA on naive CD4+; (E)CD8 on TD CD8br; (F)CD3 on TD CD4+; (G)Resting Treg %CD4; (H) CD25 on CD28+ CD4+; (I) CCR2 on myeloid DC; (J) CD33br HLA DR+ CD14dim %CD33br HLA DR+.

**Supplementary Figure 3**. Leave-one-out analysis result of PD on immunophenotypes.

(A) HLA DR+ T cell AC; (B) IgD− CD38− %B cell.

**Supplementary Figure 4**. Scatter plot of immunophenotypes on PD (Risk factor).

(A) HLA DR++ monocyte %monocyte; (B) CD45 on CD14+ monocyte; (C) CD45 on HLA DR+ T cell; (D)CD28+ DN (CD4−CD8−) %T cell; (E)Activated Treg %CD4 Treg; (F)CD39 on monocyte; (G)CD33dim HLA DR+ CD11b+ AC; (H)CD33br HLA DR+ CD14− AC. Three lines reveal the estimated effect sizes by MR methods (inverse‐variance weighted, MR-Egger and weighted median) .

**Supplementary Figure 5**. Funnel plot of immunophenotypes on PD (Risk factor).

(A) HLA DR++ monocyte %monocyte; (B) CD45 on CD14+ monocyte; (C) CD45 on HLA DR+ T cell; (D) CD28+ DN (CD4−CD8−) %T cell; (E) Activated Treg%CD4 Treg; (F) CD39 on monocyte; (G) CD33dim HLA DR+ CD11b+ AC; (H) CD33br HLA DR+ CD14− AC.

**Supplementary Figure 6**. Scatter plot of immunophenotypes on PD(Protective factor).

(A)CD16 on CD14− CD16+ monocyte; (B) CD14+ CD16+ monocyte AC; (C) CD14+ CD16+ monocyte %monocyte; (D) CD45RA on naive CD4+; (E)CD8 on TD CD8br; (F) CD3 on TD CD4+; (G) Resting Treg %CD4; (H) CD25 on CD28+ CD4+; (I) CCR2 on myeloid DC; (J) CD33br HLA DR+ CD14dim %CD33br HLA DR+. Three lines reveal the estimated effect sizes by MR methods (inverse‐variance weighted; MR-Egger and weighted median) .

**Supplementary Figure 7**. Funnel plot of immunophenotypes on PD (Protective factor).

(A) CD16 on CD14− CD16+ monocyte; (B) CD14+ CD16+ monocyte AC; (C) CD14+ CD16+ monocyte %monocyte; (D) CD45RA on naive CD4+; (E) CD8 on TD CD8br; (F) CD3 on TD CD4+; (G) Resting Treg %CD4; (H)CD25 on CD28+ CD4+; (I) CCR2 on myeloid DC; (J) CD33br HLA DR+ CD14dim %CD33br HLA DR+.

**Supplementary Table 1** MR-PRESSO analysis for the association between immune cells and PD.

| **Immune cells (exposure)** | **MR Analysis** | **Causal Estimate** | **Sd** | **T-stat** | **p-value** | **RSS_obs_** | **Global Test p-value** |
| --- | --- | --- | --- | --- | --- | --- | --- |
| ebi-a-GCST90001427 | MR-PRESSO | 0.087 | 0.040 | 2.191 | 0.037 | 28.225 | 0.554 |
| ebi-a-GCST90001448 | MR-PRESSO | 0.086 | 0.040 | 2.147 | 0.047 | 18.294 | 0.500 |
| ebi-a-GCST90001475 | MR-PRESSO | 0.102 | 0.042 | 2.460 | 0.026 | 20.927 | 0.339 |
| ebi-a-GCST90001482 | MR-PRESSO | 0.036 | 0.015 | 2.330 | 0.026 | 25.307 | 0.863 |
| ebi-a-GCST90001487 | MR-PRESSO | 0.066 | 0.024 | 2.741 | 0.010 | 21.791 | 0.874 |
| ebi-a-GCST90001518 | MR-PRESSO | 0.025 | 0.012 | 2.106 | 0.045 | 26.246 | 0.594 |
| ebi-a-GCST90001521 | MR-PRESSO | 0.064 | 0.030 | 2.157 | 0.041 | 28.124 | 0.390 |
| ebi-a-GCST90001525 | MR-PRESSO | 0.073 | 0.027 | 2.669 | 0.013 | 29.622 | 0.381 |
| ebi-a-GCST90001580 | MR-PRESSO | 0.072 | 0.034 | 2.132 | 0.046 | 19.229 | 0.654 |
| ebi-a-GCST90001585 | MR-PRESSO | 0.093 | 0.034 | 2.712 | 0.012 | 24.142 | 0.639 |
| ebi-a-GCST90001655 | MR-PRESSO | 0.092 | 0.039 | 2.340 | 0.029 | 24.271 | 0.442 |
| ebi-a-GCST90001698 | MR-PRESSO | 0.001 | 0.001 | 2.108 | 0.035 | 702.194 | 0.657 |
| ebi-a-GCST90001760 | MR-PRESSO | 0.073 | 0.033 | 2.211 | 0.040 | 21.567 | 0.447 |
| ebi-a-GCST90001822 | MR-PRESSO | 0.069 | 0.033 | 2.113 | 0.045 | 26.316 | 0.525 |
| ebi-a-GCST90001844 | MR-PRESSO | 0.060 | 0.029 | 2.093 | 0.046 | 30.427 | 0.384 |
| ebi-a-GCST90001909 | MR-PRESSO | 0.102 | 0.042 | 2.431 | 0.029 | 14.492 | 0.565 |
| ebi-a-GCST90001918 | MR-PRESSO | 0.062 | 0.029 | 2.105 | 0.053 | 16.253 | 0.551 |
| ebi-a-GCST90001962 | MR-PRESSO | 0.197 | 0.098 | 2.007 | 0.115 | 6.866 | 0.398 |
| ebi-a-GCST90001979 | MR-PRESSO | 0.049 | 0.018 | 2.773 | 0.011 | 13.791 | 0.931 |
| ebi-a-GCST90002013 | MR-PRESSO | 0.063 | 0.015 | 4.275 | 0.001 | 4.839 | 0.994 |
| ebi-a-GCST90002034 | MR-PRESSO | 0.032 | 0.013 | 2.469 | 0.022 | 14.583 | 0.907 |
| ebi-a-GCST90002057 | MR-PRESSO | 0.129 | 0.047 | 2.751 | 0.016 | 13.534 | 0.618 |
| ebi-a-GCST90002081 | MR-PRESSO | 0.100 | 0.045 | 2.230 | 0.035 | 30.382 | 0.306 |
| ebi-a-GCST90002098 | MR-PRESSO | 0.056 | 0.021 | 2.689 | 0.011 | 32.802 | 0.589 |

MR, Mendelian Randomization; PE, Preeclampsia and Eclampsia; SD, Standard Deviation; RSSobs, Observed Residual Sum of Squares; SNP.

**Supplementary Table 2** Heterogeneity and pleiotropy test (risk factor-TBNK group).

| **Exposure** | **Outcome** | **Heterogeneity** | | | | | | **Pleiotropy** | | |
| --- | --- | --- | --- | --- | --- | --- | --- | --- | --- | --- |
|  |  | **MR Egger** | | | **Inverse variance weighted** | | | **MR Egger** | | |
|  |  | ***Q*** | **Q_df** | **p-value** | ***Q*** | **Q_df** | **p-value** | **Intercept** | **SE** | **p-value** |
| HLA DR++ monocyte %monocyte | PD | 18.356 | 15 | 0.244 | 18.813 | 16 | 0.278 | -0.011 | 0.018 | 0.550 |
| CD45 on CD14+ monocyte | PD | 12.575 | 13 | 0.481 | 12.665 | 14 | 0.553 | 0.005 | 0.018 | 0.769 |
| CD45 on HLA DR+ T-cell | PD | 13.230 | 14 | 0.509 | 13.294 | 15 | 0.580 | 0.005 | 0.019 | 0.804 |

**Supplementary Table 3** Heterogeneity and pleiotropy test (risk factor-Treg group).

| **Exposure** | **Outcome** | **Heterogeneity** | | | | | | **Pleiotropy** | | |
| --- | --- | --- | --- | --- | --- | --- | --- | --- | --- | --- |
|  |  | **MR Egger** | | | **Inverse variance weighted** | | | **MR Egger** | | |
|  |  | **Q** | **Q_df** | **p-value** | **Q** | **Q_df** | **p-value** | **Intercept** | **SE** | **p-value** |
| CD28+ DN (CD4^−^CD8^−^) %T-cell | PD | 21.999 | 20 | 0.341 | 22.070 | 21 | 0.395 | 0.004 | 0.015 | 0.802 |
| Activated Treg %CD4 Treg | PD | 20.761 | 28 | 0.835 | 20.797 | 29 | 0.866 | -0.002 | 0.012 | 0.851 |
| CD39 on monocyte | PD | 13.783 | 20 | 0.841 | 13.788 | 21 | 0.879 | -0.001 | 0.012 | 0.947 |

**Supplementary Table 4** Heterogeneity and pleiotropy test (risk factor-Myeloid group).

| **Exposure** | **Outcome** | **Heterogeneity** | | | | | | **Pleiotropy** | | |
| --- | --- | --- | --- | --- | --- | --- | --- | --- | --- | --- |
|  |  | **MR Egger** | | | **Inverse variance weighted** | | | **MR Egger** | | |
|  |  | **Q** | **Q_df** | **p-value** | **Q** | **Q_df** | **p-value** | **Intercept** | **SE** | **p-value** |
| CD33dim HLA DR+ CD11b+ AC | PD | 27.861 | 25 | 0.314 | 27.869 | 26 | 0.365 | 0.001 | 0.0148 | 0.935 |
| CD33br HLA DR+ CD14^−^ AC | PD | 23.560 | 26 | 0.601 | 23.767 | 27 | 0.643 | -0.005 | 0.010 | 0.653 |

**Supplementary Table 5** Heterogeneity and pleiotropy test (protective factor-monocyte group).

| **Exposure** | **Outcome** | **Heterogeneity** | | | | | | **Pleiotropy** | | |
| --- | --- | --- | --- | --- | --- | --- | --- | --- | --- | --- |
|  |  | **MR Egger** | | | **Inverse variance weighted** | | | **MR Egger** | | |
|  |  | **Q** | **Q_df** | **p-value** | **Q** | **Q_df** | **p-value** | **Intercept** | **SE** | **p-value** |
| CD16 on CD14^−^ CD16+ monocyte | PD | 12.590 | 20 | 0.894 | 12.709 | 21 | 0.918 | 0.005 | 0.013 | 0.734 |
| CD14+ CD16+ monocyte AC | PD | 17.966 | 19 | 0.525 | 18.337 | 20 | 0.565 | 0.010 | 0.016 | 0.550 |
| CD14+ CD16+ monocyte %monocyte | PD | 19.962 | 24 | 0.699 | 22.853 | 25 | 0.586 | -0.0294 | 0.0173 | 0.102 |

**Supplementary Table 6** Heterogeneity and pleiotropy test (protective factor-T-cell maturation stage group).

| **Exposure** | **Outcome** | **Heterogeneity** | | | | | | **Pleiotropy** | | |
| --- | --- | --- | --- | --- | --- | --- | --- | --- | --- | --- |
|  |  | **MR Egger** | | | **Inverse variance weighted** | | | **MR Egger** | | |
|  |  | **Q** | **Q_df** | **p-value** | **Q** | **Q_df** | **p-value** | **Intercept** | **SE** | **p-value** |
| CD45RA on naive CD4+ | PD | 31.639 | 32 | 0.485 | 31.701 | 33 | 0.532 | 0.003 | 0.012 | 0.805 |
| CD8 on TD CD8br | PD | 12.120 | 13 | 0.518 | 12.440 | 14 | 0.571 | 0.010 | 0.018 | 0.581 |
| CD3 on TD CD4+ | PD | 27.076 | 25 | 0.352 | 27.305 | 26 | 0.393 | -0.006 | 0.013 | 0.649 |

**Supplementary Table 7** Heterogeneity and pleiotropy test (protective factor-other group).

| **Exposure** | **Outcome** | **Heterogeneity** | | | | | | **Pleiotropy** | | |
| --- | --- | --- | --- | --- | --- | --- | --- | --- | --- | --- |
|  |  | **MR Egger** | | | **Inverse variance weighted** | | | **MR Egger** | | |
|  |  | **Q** | **Q_df** | **p-value** | **Q** | **Q_df** | **p-value** | **Intercept** | **SE** | **p-value** |
| Resting Treg %CD4 | PD | 23.986 | 30 | 0.773 | 24.530 | 31 | 0.788 | -0.010 | 0.013 | 0.467 |
| CD25 on CD28+ CD4+ | PD | 4.967 | 3 | 0.174 | 5.167 | 4 | 0.271 | 0.019 | 0.056 | 0.751 |
| CCR2 on myeloid DC | PD | 3.998 | 12 | 0.983 | 4.269 | 13 | 0.988 | -0.008 | 0.015 | 0.613 |
| CD33br HLA DR+ CD14dim %CD33br HLA DR+ | PD | 24.275 | 23 | 0.389 | 25.141 | 24 | 0.398 | -0.012 | 0.013 | 0.374 |

**Supplementary Table 8** Heterogeneity and pleiotropy test (PD on immune cell phenotype).

| **Exposure** | **Outcome** | **Heterogeneity** | | | | | | **Pleiotropy** | | |
| --- | --- | --- | --- | --- | --- | --- | --- | --- | --- | --- |
|  |  | **MR Egger** | | | **Inverse variance weighted** | | | **MR Egger** | | |
|  |  | **Q** | **Q_df** | **p-value** | **Q** | **Q_df** | **p-value** | **Intercept** | **SE** | **p-value** |
| PD | HLA DR+ T-cell AC | 7.472 | 13 | 0.876 | 8.352 | 14 | 0.870 | -0.022 | 0.023 | 0.365 |
| PD | IgD−CD38^−^ %B-cell | 16.332 | 13 | 0.232 | 16.894 | 14 | 0.262 | -0.018 | 0.027 | 0.515 |
